# Editorial: Deciphering the T cell response in SARS-CoV-2 infection

**DOI:** 10.3389/fimmu.2024.1419167

**Published:** 2024-07-10

**Authors:** Laura Maggi, Alessio Mazzoni, Sara De Biasi

**Affiliations:** ^1^ Department of Clinical and Experimental Medicine, School of Human Health Sciences, University of Florence, Firenze, Italy; ^2^ Department of Medical and Surgical, Maternal-Infantile and Adult Sciences, University of Modena and Reggio Emilia, Modena, Italy

**Keywords:** T cells, immunological memory, SARS-CoV-2, vaccine, COVID-19

COVID-19 is an infectious disease emerged in 2020, caused by the novel coronavirus SARS-CoV-2. The immune system has a primary role in pathogen elimination and a rapid and effective response can limit disease severity. In this context, T cells play the major role in cell mediated adaptive immune response.

The aim of this Research Topic was to collect and build a comprehensive and specific consensus signature of T cell immunity in SARS-CoV-2 infection as well as following vaccination. In particular, twenty-three articles have been published under this Research Topic including Brief Research Reports, Original Researches and Reviews, covering the topic of the issue.

The characterization of T cell response, including both the polyclonal and the SARS-CoV-2 specific response, is crucial to define the status of the immune response at different stages of COVID-19, it can be important for improving disease diagnosis and it may represent a possible parameter for monitoring the efficacy of therapeutical treatments. Severe COVID-19 patients were characterized by an increase of exhausted and terminal differentiated CD8+ T cells (Mortezaee and Majidpoor; Schreibing et al.) and by a reduction of circulating Th1 and Treg cells: the impaired frequency of the latter at baseline may predict a clinical worsening during hospitalization (Caldrer et al.). In the case of mild-moderate COVID-19, SARS-CoV-2 specific T cells were reduced but detectable up to 10 months after symptoms onset in both adults and children, even if lower in the latter cohort (Kaaijk et al.), whereas a specific set of V-J gene combination has been associated to asymptomatic and re-detectable positive cases, different from symptomatic ones (Li et al.). Cytokine storm plays a major role in the immunopathogenesis of severe COVID-19 suggesting the use of immunomodulatory drugs in association with antivirals as treatments for COVID-19 and leading to a downregulation of pro-inflammatory cytokines (Martonik et al.). Interestingly, the levels of pro-inflammatory cytokines during the infection phase could predict the response to Tocilizumab (anti-IL6R) treatment (De Biasi et al.). Instead, the entity of CD4+ and CD8+ T cells immune disfunction described at 6 months from severe COVID-19 correlated with the development/persistence of long COVID symptoms (Wiech et al.), even if these clinical manifestations are not strictly related to severe COVID-19 group and all infected patients could develop long COVID-19 despite initial T cells dysfunction.

In the first wave of COVID-19 pandemic, the different grade of disease may have been partially influenced by pre-existing memory T cells directed against other coronaviruses and cross reactive to SARS-CoV-2, found mainly in bone marrow but also in blood of unexposed subjects, derived by cross-reaction with other antigens (Eggenhuizen et al.; Eggenhuizen et al.; Li et al.) and potentially restricted to specific HLA class II allotypes (Hyun et al.). In the subsequent waves of COVID-19 the clinical course of the disease was mainly impacted by emerged viral variants and by vaccination campaign. Mutations occurring on the different SARS-CoV-2 variants evolved after Wuhan strain, affected mainly the neutralizing activity of specific antibodies, whereas immunological memory mediated by specific CD4+ and CD8+ T cells was highly conserved (Boni et al.; Isaeva et al.; Mazzoni et al.). The characterization of the T cell response can be very informative to decipher SARS-CoV-2-specific T cells associated with viral clearance but also useful for vaccine development and for planning vaccination strategies. Many studies supported the concept that anti-SARS-CoV-2 vaccine is able to induce anti-Spike adaptive immune response in healthy subjects with a persistence of Spike specific IgG serum levels and circulating CD4+ and CD8+ T cells up 7 months after two-dose mRNA vaccine (Vitiello et al.) and that the booster dose efficiently increased them (Kurt et al.). Similar data were obtained also in cohort of patients with autoimmune rheumatic and musculoskeletal disease, even if the entity of immunogenicity was reduced in case of B-cell depleting therapy; however, these data demonstrated the safety of vaccination and the importance to promote it also in these subjects to prevent severe COVID-19 (Szebeni et al.). Interestingly, SARS-CoV- 2-specific adaptive immune response induced by vaccine was described to be in part different form that induced by natural infection mainly in terms of Th1 polarization and highly polyfunctional T cells population in the former ones (Lo Tartaro et al.). Improving vaccines design and vaccination strategy could derive from: 1) studies based on vaccinome approach leading to identification of antigenic epitopes selected from in-silico models of immune response prediction and to design a multi-epitope subunit vaccine (Khan et al.); 2) investigation of hypo-responsiveness to infections and vaccination in patients characterized by chronic immune activation leading to CD4+ T cells lymphopenia (Wolday et al.).

Even if the enormous number of subjects infected with SAR-CoV-2 and/or vaccinated allowed to conduct most of SARS-CoV-2 related immunological studies on human samples, COVID-19 mouse models lead to identification of immunogenic peptides of Spike and nucleocapsid protein for CD4+ T cells by MHC II tetramers (Bricio-Moreno et al.). Moreover, these results paved the way to use COVID-19 mouse models to further investigate the features of adaptive T cells immune response for human translational applications.

In conclusion, this Research Topic, with a variety of articles, has provided novel insight on different aspects of the role T cells mediated immune response in case of COVID19 or SARS-CoV-2 vaccination, with promising outcomes and future perspectives. As Editors, we would like to thank all the contributing authors and the people in Frontiers in Immunology for their support and to give us the opportunity to manage this Research Topic.

## Author contributions

LM: Writing – original draft. AM: Writing – original draft. SB: Writing – original draft.

